# Efficacy of Immune Checkpoint Inhibitors vs. Tyrosine Kinase Inhibitors/Everolimus in Adjuvant Renal Cell Carcinoma: Indirect Comparison of Disease-Free Survival

**DOI:** 10.3390/cancers16030557

**Published:** 2024-01-28

**Authors:** Andrea Ossato, Lorenzo Gasperoni, Luna Del Bono, Andrea Messori, Vera Damuzzo

**Affiliations:** 1 Department of Pharmaceutical and Pharmacological Sciences, University of Padua, 35131 Padova, Italy; andrea.ossato@studenti.unipd.it; 2 Oncological Pharmacy Unit, IRCCS Istituto Romagnolo per lo Studio dei Tumori (IRST) “Dino Amadori”, 47014 Meldola, Italy; lorenzo.gasperoni@irst.emr.it; 3 Azienda Ospedaliera Universitaria Pisana, 56100 Pisa, Italy; lunadelbono@gmail.com; 4 HTA Unit, Regional Health Service, 50139 Florence, Italy; 5 Hospital Pharmacy, Vittorio Veneto Hospital, 31029 Vittorio Veneto, Italy; 6 Italian Society of Clinical Pharmacy and Therapeutics (SIFaCT), 10123 Turin, Italy

**Keywords:** indirect comparison, Shiny method, reconstructed individual patient data, disease-free survival, renal cell carcinoma, adjuvant setting

## Abstract

**Simple Summary:**

The proven efficacy of mTOR inhibitor (mTORI), tyrosine kinase inhibitor (TKI) or immune checkpoint inhibitor (ICI) therapies in metastatic renal cell carcinoma (RCC) suggests that these agents should be investigated as adjuvant therapy with the aim of eliminating undetectable microscopic residual disease after curative resection. Our study aimed to compare the efficacy of these treatments using an innovative method that reconstructs individual patient data from Kaplan–Meier (KM) curves. Nine phase III trials describing different treatment options for adjuvant RCC were selected. Individual patient data were reconstructed from KM curves of disease-free survival (DFS) using the IPDfromKM method. DFS was then compared between the combination treatments and the control arm (placebo). The results were summarized as multi-treatment KM curves. Standard statistical tests were used, including the hazard ratio for superiority and the likelihood ratio test for heterogeneity. In the population of these nine trials, our study showed that two ICIs (nivolumab plus ipilimumab and pembrolizumab) and one TKI (sunitinib) were superior to the placebo, whereas the remaining TKIs and mTORIs were not. As we assessed DFS as the primary endpoint for the adjuvant comparison, the overall survival benefit remains unknown. This novel approach to studying survival has allowed us to make all of the indirect head-to-head comparisons between these agents in a context where no “real” comparative trials have been conducted.

**Abstract:**

Background: The proven efficacy of mTOR inhibitors (mTORIs), tyrosine kinase inhibitors (TKIs) or immune checkpoint inhibitors (ICIs) in metastatic renal cell carcinoma (RCC) suggests that these agents should be investigated as adjuvant therapy with the aim of eliminating undetectable microscopic residual disease after curative resection. The aim of our study was to compare the efficacy of these treatments using an innovative method of reconstructing individual patient data. Methods: Nine phase III trials describing adjuvant RCC treatments were selected. The IPDfromKM method was used to reconstruct individual patient data from Kaplan–Meier (KM) curves. The combination treatments were compared with the control arm (placebo) for disease-free survival (DFS). Multi-treatment KM curves were used to summarize the results. Standard statistical tests were performed. These included hazard ratio and likelihood ratio tests for heterogeneity. Results: In the overall population, the study showed that two ICIs (nivolumab plus ipilimumab and pembrolizumab) and one TKI (sunitinib) were superior to the placebo, whereas both TKIs and mTORIs were inferior. As we assessed DFS as the primary endpoint for the adjuvant comparison, the overall survival benefit remains unknown. Conclusions: This novel approach to investigating survival has allowed us to conduct all indirect head-to-head comparisons between these agents in a context where no “real” comparative trials have been conducted.

## 1. Introduction

Renal cell carcinoma (RCC) is the most common type of kidney cancer, accounting for about 2% of all cancer diagnoses worldwide. Approximately 80% of RCCs are clear cell tumors [1].

Over the last 20 years, the prognosis for patients with metastatic RCC has improved thanks to the results of clinical trials with mTOR inhibitors (mTORIs), tyrosine kinase inhibitors (TKIs) or immune checkpoint inhibitors (ICIs). The most recent revolution is the treatment of metastatic RCC with ICI combinations or ICI–TKI combinations [2,3,4,5], which has resulted in a significant improvement in survival, although not a cure.

Prevention of metastatic disease remains a priority in the curative setting of early stage RCC. For patients with locoregional RCC, partial or radical nephrectomy is the standard of care, and adjuvant treatment is an option to reduce the risk of recurrence, considering that 40% of surgically resected patients with stage II–III disease will relapse [6,7,8,9,10].

The proven efficacy of ICI, TKI and mTORI therapies in metastatic RCC suggested that these agents should be investigated as adjuvant therapy with the aim of eliminating any residual undetectable microscopic disease after curative resection.

Numerous randomized phase III trials in the adjuvant treatment of patients with RCC have ended with conflicting results, but overall, they seem to show a greater benefit of treatment with ICIs compared to TKIs and mTORIs [11,12,13,14,15,16,17,18,19]. As a result of this uncertainty, adjuvant therapies have also had different regulatory pathways. In fact, sunitinib, the first adjuvant therapy approved by the Food and Drug Administration (FDA) for the adjuvant treatment of RCC, was not approved by the European Medicines Agency (EMA).

In this scenario, given the available results and the lack of head-to-head comparisons between the adjuvant treatment options studied to date, we conducted an analysis using a new artificial intelligence technique (called the “IPDfromKM” method or Shiny method) to compare disease-free survival (DFS) in patients with resected primary RCC at risk of recurrence. Only results from phase III randomized clinical trials (RCTs) with adjuvant mTORI or ICI or TKI were included in the analysis.

The IPDfromKM method is a new artificial intelligence tool that reconstructs individual patient data from the graph of Kaplan–Meier (KM) curves and allows cross-study comparisons to be made based on reconstructed patients [20,21]. This is a relatively new method for generating new original clinical evidence and is particularly suitable for indirect comparison of time-to-event endpoints, especially those with a long follow-up, because it takes into consideration the time at which events occurred, while a standard binary meta-analysis ignores this information. In addition, the IPDfromKM method presents an easy-to-understand summary of results generating a unique KM chart containing the curves based on reconstructed patients and pooling all patients who received the same treatment, regardless of the clinical trial. In other words, the Forest plot typical of standard binary meta-analysis is replaced by a survival plot with as many KM curves as the number of treatments compared. The main treatment regimens can be further compared with each other using standard statistical methods such as the hazard ratio. Thanks to this method, in the present report we provide a comparative overview of the main adjuvant treatments available for RCC patients and determine their place in therapy and their relative efficacy.

## 2. Materials and Methods

### 2.1. Study Design

In accordance with recent publications [5,21,22], we conducted a comprehensive literature review to identify primary treatment options for adjuvant treatment of RCC. Following the selection of relevant studies, we utilized the IPDfromKM method to reconstruct individual patient data from KM graphs and consequently perform head-to-head indirect comparisons between treatments [20]. Our analysis focused on disease-free survival (DFS) as the primary endpoint. Results were shown through multi-treatment KM curves.

### 2.2. Literature Search

We searched the ClinicalTrials.gov database to identify randomized controlled trials (RCTs) that were eligible for our analysis (last search on 15 November 2023). The following search terms were used: “renal cell carcinoma” OR “RCC”. Only phase III randomized interventional clinical trials were selected applying the filter option. By eliminating duplicates, 146 RCTs emerged. The main inclusion criteria were: (a) RCC treatment (not diagnostic, imaging or surgical trials, not other diseases or solid tumors of different origin); (b) adjuvant treatment (not neoadjuvant/adjuvant treatment, not perioperative treatment, not locally advanced or metastatic treatment); (c) ICI or TKI or mTORI treatment; (d) inclusion of non-metastatic patients; (e) DFS endpoint; and (f) publication of results as a KM curve. The selection of articles from our literature search was based on the PRISMA algorithm, which recorded the reasons for inclusion and exclusion of each trial [23]; the final list of included trials was determined in the last step of the PRISMA flow.

For each trial included, we recorded the number of patients enrolled and the number of events (defined as first documented local or distant recurrence of RCC, secondary systemic malignancy, or death from any cause, whichever occurred first). To avoid duplicate inclusion of patients from the same trial, only the most recent publication was included.

### 2.3. Reconstruction of Individual Patient Data

Patient-level data were reconstructed from KM curves using the IPDfromKM method, as previously described [20,21]. The curves were digitized with Webplot digitizer (version 4.5 online; URL https://apps.automeris.io/wpd/, accessed on 10 January 2024) and subsequently entered into the individual patient data reconstruction tool of the Shiny software (version 1.2.3.0).

Reconstructed individual patient data included observation time (defined as difference between enrolment and last follow-up) and patient outcome at last follow-up (alive, dead, censored).

### 2.4. Statistical Analysis

Restricted mean survival and DFS were estimated for each experimental treatment in comparison to placebo using Cox statistics for time-to-event endpoints; hazard ratio (HR) with 95% confidence interval (CI) and medians with 95% CI were also calculated. The likelihood ratio test was used to assess heterogeneity in outcomes between control groups of different RCTs. Moreover, indirect comparisons between treatments were assessed using the Cox model through four specific R-platform (version 4.2.1) packages for statistical analyses: survival, survRM2, survminer and ggsurvplot (2020; https://www.R-project.org/, accessed on 18 December 2023).

## 3. Results

**Figure 1:**
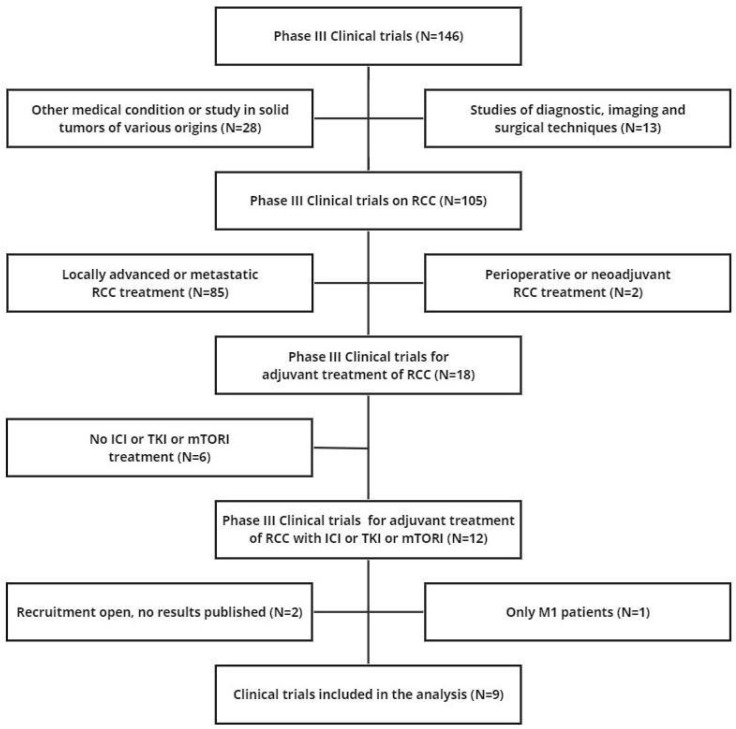
PRISMA flowchart of the process of trial selection. Abbreviations: RCC, renal cell carcinoma; DFS, disease-free survival.

Nine trials met the criteria for inclusion in our analysis (see Figure 1 for the PRISMA flowchart and Table 1 for RCT characteristics). Three trials involved an experimental treatment with parenteral ICIs while six trials were based on oral therapies such as TKIs (n = 5) or mTORI (n = 1). In all nine trials, the control arm was the placebo. In the application of the IPDfromKM method, we reconstructed 20 patient cohorts which represented the clinical material to perform our indirect comparisons.

**Figure 2:**
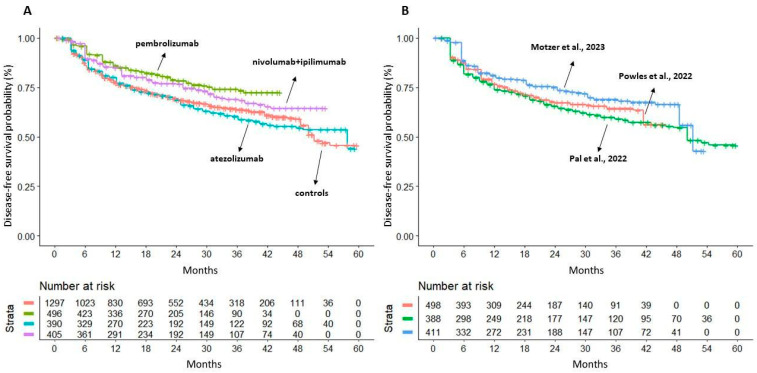
Panel (**A**) Shiny technique applied to placebo (n = 1297 from three trials; in red) and to three combination treatments: (a) pembrolizumab 200 mg (n = 496; in green); (b) atezolizumab 12,000 mg (n = 390; in light blue); (c) nivolumab 240 mg + ipilimumab 1 mg/kg (n = 405; in purple). Panel (**B**) Kaplan–Meier curves generated after reconstructing patient-level data from the three control arms of the included trials (placebo treated): Powles et al., 2022 (n = 498; in red [11]); Pal et al., 2022 (n = 388; in green [12]); Motzer et al., 2023 (n = 411; in blue [13]). Endpoint: disease-free survival (DFS), time in months. Abbreviations: n, number of patients.

To conduct the indirect comparisons between the three ICI treatments, the DFS KM curves from the reconstructed patients of the ICI trials were plotted individually and reported in a single multi-treatment graph, with the three placebo cohorts pooled into a single graph. In this way, a total of four curves were generated (Figure 2A). Our DFS analysis on these reconstructed patients demonstrated superiority, in comparison with the placebo, as adjuvant therapy for both pembrolizumab (HR 0.62; 95% CI 0.50–0.78; *p* < 0.001) and nivolumab plus ipilimumab (HR 0.74; 95% CI 0.60–0.93; *p* = 0.008). Atezolizumab showed no advantage in DFS compared to the placebo (HR 1.04; 95% CI 0.86–1.26; *p* = NS).

The likelihood ratio test carried out on control arms showed no heterogeneity between these cohorts (likelihood ratio test, 5.01 on 2 df, *p* < 0.08; Figure 2B).

**Figure 3:**
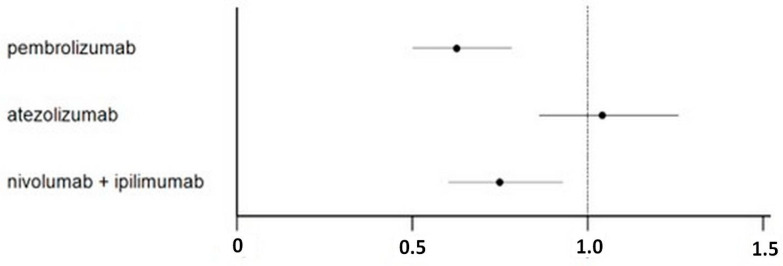
Forest plot showing the disease-free survival of adjuvant renal cell carcinoma patients treated with ICI treatment. Values are reported as HR of disease-free survival compared with controls.

Detailed results of indirect comparisons of the three ICIs treatments in all head-to-head combinations are reported as Forest plots of HRs with 95% CI (Figure 3 and Table S1). This analysis shows that pembrolizumab (HR = 0.60; 95% CI 0.45–0.81; *p* < 0.001) and nivolumab plus ipilimumab (HR = 0.72; 95% CI 0.54–0.96; *p* = 0.024) were superior to atezolizumab; on the other hand, pembrolizumab was not significantly superior to nivolumab plus ipilimumab (HR = 0.83; 95% CI 0.61–1.14; *p* = NS).

**Figure 4:**
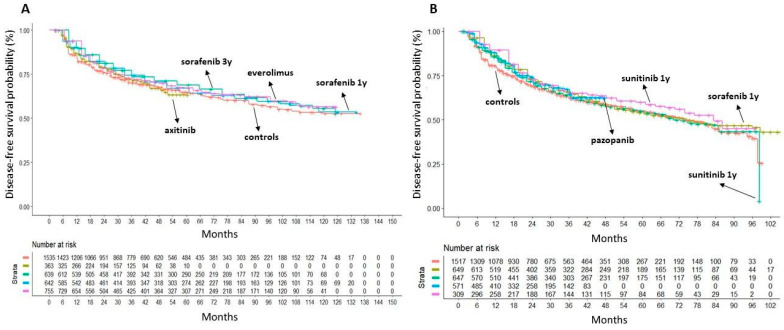
Application of the Shiny method in our subgroup analysis. Panel (**A**): comparison of DFS of the placebo (n = 1535 from ATLAS, SORCE and EVEREST trials; in red) with treatment arms of ATLAS ((axitinib n = 363; in gold); [14]), SORCE ((sorafenib for 3 years; n = 639; in turquoise) and (sorafenib for one year; n = 642; in blue); [15]) and EVEREST RCT ((everolimus; n = 755; in purple); [19]). Panel (**B**): comparison of DFS of placebo (n = 1517 from ASSURE, PROTECT and S-TRAC trials; in red) with treatment arms of ASSURE (sorafenib for 1 year (n = 649; in gold) and (sunitinib; n = 647; in light green); [16]), PROTECT (pazopanib (n = 571; in blue); [17]) and S-TRAC RCT ((sunitinib; n = 309; in purple); [18]). End-point: disease-free survival (DFS), time in months. Abbreviations: n, number of patients.

To conduct indirect comparisons across oral treatments, the DFS KM curves from reconstructed patients of TKI and mTORI trials were plotted separately and reported as a single multi-treatment graph. The six control arms of oral therapy treatments demonstrated significant heterogeneity (Figure S1). Our head-to-head indirect comparisons were designed as follows. Firstly, we identified two non-heterogeneous subgroups among these trials because patients in the ATLAS trial were comparable to those in SORCE and EVEREST trials (subgroup #1) while patients in the ASSURE trial were comparable to those in PROTECT and S-TRAC trials (subgroup #2). Likewise, patients treated with the placebo in each of these two subgroups were combined to form two separate control cohorts. Finally, the DFS of the three active arms within each subgroup was compared to that of the respective pooled controls (Figure 4).

Most oral drug treatments did not show any DFS benefit over the placebo. Only two studies with sorafenib or sunitinib demonstrated a DFS advantage, but the results were somewhat contradictory. In fact, in the SORCE trial, patients receiving sorafenib 400 mg BID for 3 years showed a significantly longer DFS than the controls (HR = 0.83; 95% CI 0.71–0.98; *p* = 0.028) but not those treated for one year (HR = 0.89; 95% CI 0.76–1.04; *p* = NS). Conversely, in the ASSURE study, patients treated with sorafenib for approximately one year (54 weeks) showed a significantly longer DFS than placebo (HR = 0.85; 95% CI 0.74–0.99; *p* = 0.036). In the S-TRAC study, sunitinib determined a significant advantage over the placebo (HR = 0.81; 95% CI 0.66–0.99; *p* = 0.041), but in the ASSURE study the sunitinib treatment arm did not confirm this result, even though the dosage was the same.

Based on these intermediate results of our analysis, in the trials in which the active arm reported a DFS benefit over placebo (namely, KEYNOTE-564, CheckMate-914, S-TRAC, SORCE, based on the advantage at 3 years in sorafenib arm, and ASSURE, based on the advantage at 1 year in sorafenib arm), the treatment arms were indirectly compared with one another in all combinations (Table S2). At the same time, we performed the heterogeneity test on the control arms (Figure S2). With a likelihood ratio test of 34.42 on 4 df, *p* < 0.001, the SORCE trial proved to be an outlier compared with the other control arms and, therefore, was excluded from the indirect comparison. After this exclusion of the SORCE study, the analysis of the control arms showed no heterogeneity (likelihood ratio test = 3.68 on 3 df, *p* = 0.3).

**Figure 5:**
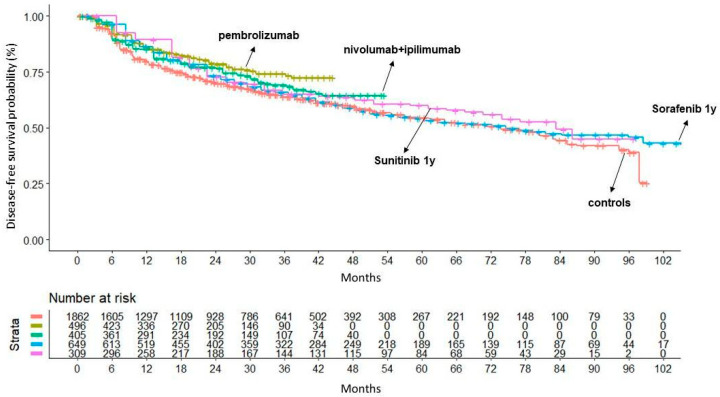
Comparison between DFS of the placebo (n = 1862 from 4 trials; in red) and of four treatments that showed survival benefit compared to control: (a) pembrolizumab (n = 496; in gold); (b) nivolumab + ipilimumab (n = 405; in light green); (c) sorafenib for 1 year (n = 649; in blue) and (d) sunitinib (n = 309; in purple). Endpoint: disease-free survival (DFS), time in months. Abbreviations: n, number of patients.

**Figure 6:**
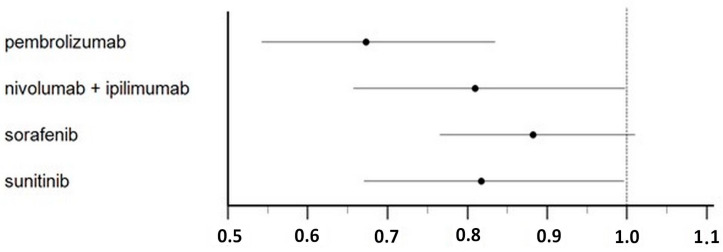
Forest plot showing the disease-free survival of adjuvant renal cell carcinoma patients treated with ICI and TKI regimens. Values are reported as HR of disease-free survival compared with controls.

Figure 5 shows the results of our main analysis in which pembrolizumab (HR 0.67; 95% CI 0.54–0.83; *p* < 0.001), nivolumab plus ipilimumab (HR 0.81; 95% CI 0.66–0.99; *p* = 0.05) and sunitinib (HR 0.82; 95% CI 0.67–0.99; *p* = 0.05) demonstrated superiority compared to the placebo. These three treatments were indirectly compared with one another, and these comparisons showed that pembrolizumab was significantly superior to 1-year treatment with sorafenib (HR = 0.76; 95% CI 0.60–0.98; *p* = 0.038); no significant difference was observed in the remaining comparisons (Figure 6, Table S3).

Overall, the efficacy of adjuvant treatments for RCC ranks as follows: (1) pembrolizumab; (2) nivolumab + ipilimumab; (3) sunitinib.

In our estimation of absolute outcome parameters, only the S-TRAC trial had a sufficient follow-up to reach the median DFS; hence, we decided to calculate the Restricted Mean Survival Times (RMSTs) as an alternative to medians. Our RMST analysis was truncated at 44 months; its results are reported in Table S3. All the five active treatments showed a DFS benefit compared to the placebo. In terms of clinical relevance, pembrolizumab produced a three-month advantage over the placebo (RMST of 36.09 vs. 32.9 months, respectively), while it was only slightly superior to the other treatments.

## 4. Discussion

The present study investigated the main treatments for adjuvant RCC using an innovative tool to reconstruct individual patient data, known as the “IPDfromKM” or “Shiny method”. This technique, applied to indirect comparisons based on standard statistics, is a valid alternative to network meta-analysis, mainly because of its ability to adjust for the different lengths of follow-up in the included trials. For this reason, the IPDfromKM method is particularly suitable for studies in the field of oncology and hemato-oncology [22], and its use has recently been extended to cardiology, where long follow-up is also common [24,25].

Additional advantages of the IPDfromKM method are the straightforward fitting procedure and the summary of results expressed as a KM curves of immediate visual interpretation. The technique also comes with some limitations like the great dependence on availability of KM curves in the original publication of the trial, which often impairs subgroup analysis, a certain dependence on the accuracy of graph processing and the absence of statistical techniques for analyzing variability such as sensitivity analysis. Nevertheless, validation studies document that, in using the Shiny method, the agreement between KM curves based on the real patients and KM curves based on reconstructed patients is excellent [26,27].

Our study is the first to indirectly compare the DFS of different drug classes in the adjuvant treatment of RCC. Our results show that two ICIs (pembrolizumab and nivolumab plus ipilimumab) and one TKI (sunitinib) showed superiority over the placebo, whereas both TKI and mTORI did not.

The pattern of heterogeneity estimated in the included trials is an interesting finding of our study. For the ICI trials, despite some differences in patient inclusion criteria (e.g., the KEYNOTE-564 and IMmotion010 trials enrolled 6% and 14% of M1 patients, respectively), the heterogeneity test showed substantial comparability between these populations. In contrast, the analysis of the TKI or mTORI trials showed significant heterogeneity in the control arms, which negatively affected the reliability of our indirect comparisons. This heterogeneity was likely due to differences in patient selection criteria, such as the presence of a high proportion of patients with early-stage tumors, sarcomatoid features or specific factors that increase the risk of relapse.

Patients at high risk of relapse should be the focus of adjuvant trials, as currently recommended [28,29], and indeed the benefit of adjuvant TKI treatment may be greater in patients at higher risk, as demonstrated in the S-TRAC trial [18] with sunitinib. In addition to eligibility criteria, the efficacy of TKIs and mTORIs may be related to other factors that influence drug exposure, such as patient adherence, discontinuation due to adverse drug reactions, different dose reduction schedules and duration of treatment. For example, in the sunitinib arm of the ASSURE trial, the dose was allowed to be reduced to 25 mg, and midway through the trial the starting dose was changed from 50 mg to 37.5 mg (a dose level not allowed in the S-TRAC trial). In addition, the duration of oral treatment was quite heterogeneous, as ATLAS trial and the sorafenib arm in the SORCE trial proposed 3 years of treatment, compared with 1 year in the other trials. Furthermore, grade 3–4 adverse events occurred in 46% of patients receiving everolimus and 49 to 72% in the TKI trials, which is more frequent than in the adjuvant ICI trials. The occurrence of adverse events may require discontinuation of treatment due to intolerance, but this may also be related to patient choice. Indeed, patients who have undergone nephrectomy are considered disease-free and may be less willing to accept serious adverse events and reduced quality of life [30]. Finally, it is possible that the vascular endothelial growth factor pathway, which is primarily targeted by TKIs, is less involved in the growth of early stage RCC, while remaining a hallmark of metastatic disease.

Overall, heterogeneity may be intrinsic and related to eligibility criteria or it may be related to less measurable variables related to the active treatment (e.g., adherence to therapy, duration of treatment, dose reduction schedule, occurrence of ADRs). In our study, we mitigated the first source of heterogeneity by performing the likelihood ratio test and comparing only studies whose placebo arms did not show heterogeneity.

Given that life expectancy after nephrectomy is nearly 40% at 10 years [29,30], a limitation of the current analysis is that OS was not investigated. Indeed, most of the studies included in the analysis had DFS as primary endpoint and OS as secondary endpoint. The availability of OS results is certainly an advantage as it is a simple endpoint, reliable to measure, easy to interpret and of great clinical utility. However, OS analyses require long follow-up times, and results may be affected by non-cancer deaths and subsequent lines of therapy. Further follow-up and maturation of OS data might influence the conclusions drawn from the study. However, even if DFS benefit may not translate into an OS benefit, a longer DFS will certainly postpone the initiation of therapies for the advanced setting which require greater intensity of care and lead to greater patient involvement and reduced quality of life. We accepted DFS as the primary endpoint for the adjuvant comparison, but, as the benefit in OS remains unknown, it is an open question whether the DFS advantage is sufficient to support the financial burden of these therapies. In addition to these economic issues, further follow-up of these trials will show whether there are long-term survivors and whether the superiority of pembrolizumab over other TKIs is confirmed. Patient perception can contribute to a more complete picture of the patient experience of adjuvant therapy, although the heterogeneity of patient selection remains a limitation. The patient-reported outcome (PRO) analysis in the ASSURE trial highlighted that sunitinib was associated with significant fatigue compared to sorafenib treatment or the placebo [31]. The PRO analysis in the S-TRAC trial showed that adjuvant sunitinib therapy was not associated with clinically meaningful deterioration in most quality-of-life measures, with the exceptions of PRO scores for diarrhea and loss of appetite, which reached a clinically meaningful difference [32]. Adjuvant pembrolizumab therapy in KEYNOTE-564 did not compromise patient health-related quality of life [33]. Finally, the combination of clinical findings from the adjuvant RCC treatment trials with artificial intelligence tools (e.g., IPDfromKM) and molecular insights from the machine learning study may lead to a more comprehensive understanding of RCC biology, its impact on treatment outcomes and treatment efficacy. These techniques may lead to new hypotheses for future research and contribute to the development of more effective and individualized treatment strategies [34].

## 5. Conclusions

In conclusion, while waiting for data with longer follow-up, most adjuvant treatments for RCC are currently approved, and the oncologist is faced with difficult clinical decisions. Our study, despite the limitations described above, mostly related to heterogeneity issues, allowed head-to-head comparisons between different adjuvant regimens for RCC, ranking their efficacy and showing pembrolizumab as the most effective option.

## Figures and Tables

**Table 1:** Main information about the nine RCTs included in the analysis.

#	Trial	Reference	Treatments under Comparison	DFS	
				Treatment Group (Events/Patients)	Controls (Events/Patients)
ARCC01	KEYNOTE-564 (two-arm)	Powles et al. [11]	Pembrolizumab (200 mg) Q3W (max 17 cycles); Placebo Q3W.	114/496	169/498
ARCC02	IMmotion010 (two-arm)	Pal et al. [12]	Atezolizumab (1200 mg) Q3W (max 16 cycles); Placebo Q3W.	164/390	168/388
ARCC03	CheckMate914 (two-arm)	Motzer et al. [13]	Nivolumab (240 mg) Q2W (max 12 cycles) + Ipilimumab (1 mg/kg) Q6W (max 4 cycles); Placebo Q2W + Q6W.	110/405	118/411
TKI01	ATLAS (two-arm)	Gross-Goupil et al. [14]	Axitinib (5 mg) BID (max 3 years); Placebo BID (max 3 years).	96/363	107/361
TKI02	SORCE (three-arm)	Eisen et al. [15]	Sorafenib (400 mg) BID for 3 years	245/639	167/430
			Sorafenib (400 mg) BID for 1 year plus placebo for 2 years; Placebo BID for 3 years.	242/642	
TKI03	ASSURE (three-arm)	Haas et al. [16]	Sunitinib (50 mg) (4 weeks on/2 off) for 54 weeks;	284/647	287/647
			Sorafenib (400 mg) BID for 54 weeks; Placebo for 54 weeks.	284/649	
TKI04	PROTECT (two-arm)	Motzer et al. [17]	Pazopanib (600 mg) for 1 year; Placebo for 1 year.	194/571	202/564
TKI05	S-TRAC (two-arm)	Ravaud et al. [18]	Sunitinib (50 mg) (4 weeks on/2 off) for 52 weeks; Placebo for 52 weeks.	113/309	144/306
mTORI01	EVEREST (two-arm)	Ryan et al. [19]	Everolimus (10 mg) for 54 weeks; Placebo for 54 weeks.	262/755	294/744

## Data Availability

The data presented in this study are available in the article and in the Supplementary Materials.

## Supplementary Materials

The following supporting information can be downloaded at: https://www.mdpi.com/article/10.3390/cancers16030557/s1, Figure S1: Visual representation of indirect treatment comparisons of six control arms of oral therapy treatments; Figure S2: Visual representation of indirect treatment comparisons for DFS of control arms of KEYNOTE-564, CheckMate-914, S-TRAC, SORCE and ASSURE Trials. Table S1: HR of inter-treatment comparison between the three ICIs treatments; Table S2: HR of inter-treatment comparison between adjuvant treatments for RCC; Table S3: Restricted mean survival time (RMST) of the five active treatments that showed a DFS benefit compared to the placebo. References [11,13,14,15,16,17,18,19] are cited in the Supplementary Materials.
