# Supplementary material for: Efficacy of Immune Checkpoint Inhibitors vs. Tyrosine Kinase Inhibitors/Everolimus in Adjuvant Renal Cell Carcinoma: Indirect Comparison of Disease-Free Survival
Source: Cancers (Basel). 2024 Jan 28;16(3):557. doi: 10.3390/cancers16030557 (PMC10854775; doi:10.3390/cancers16030557)
Supplement: Supplementary file 1 [file cancers-16-00557-s001.zip › cancers-2806317-supplementary.pdf]

Efficacy of Immune Checkpoint Inhibitors *vs* Tyrosine Kinase Inhibitors/Everolimus in Adjuvant Renal Cell Carcinoma: Indirect Comparison of Disease-Free Survival

by Andrea Ossato, Lorenzo Gasperoni, Luna Del Bono, Andrea Messori, and Vera Damuzzo

SUPPLEMENTARY MATERIAL

Figure S1. After reconstruction of individual patient data from control arms of included trials (placebo treatment), the following Kaplan-Meier curves were generated from: Gross-Goupil et al., 2018 (n=361; in red [1]); Eisen et al., 2020 (n=430; in gold [2]); Haas et al., 2016 (n=647; in green [3]); Motzer et al., 2017 (n=564; in light blue [4]); Ravaud et al., 2016 (n=306; in blue [5]) and Ryan et al., 2023 (n=744; in purple [6]). N: number of enrolled patients. Endpoint: disease-free survival (DFS), Time in months. See the reference list at the end of this file.

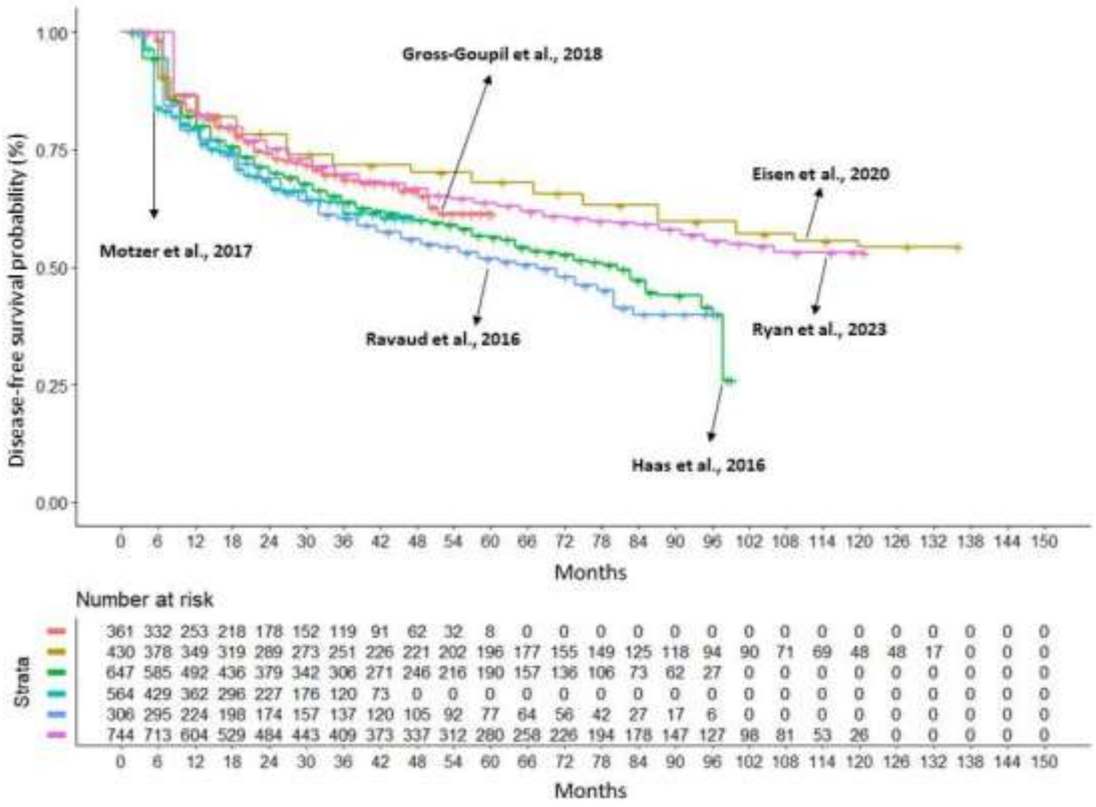

Figure S2. After reconstruction of individual patient data from control arms of included trials (placebo treatment), the following Kaplan-Meier curves were generated from: Powles et al., 2022 (n=498; in red [7]); Motzer et al., 2023 (n=411; in gold [8]); Eisen et al., 2020 (n=430; in green [2]); Haas et al., 2016 (n=647; in light blue [3]); and Ravaud et al., 2016 (n=306; in purple [5]). N: number of enrolled patients. Endpoint: disease-free survival (DFS), Time in months. See the reference list at the end of this file.

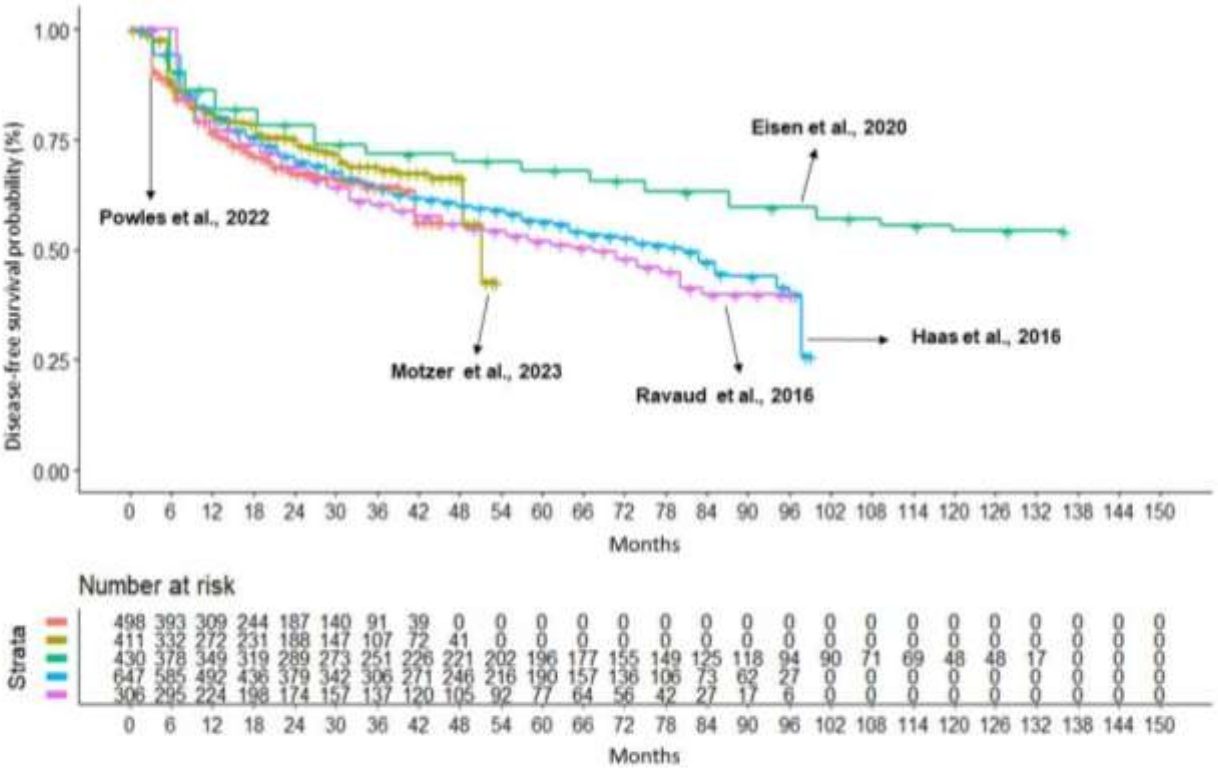

Table S1. Head-to-head indirect comparisons between ICIs.

DFS	HR	Lower.95	Upper.95
Pembrolizumab	0.6269	0.5019	0.7829
Atezolizumab	1.042	0.8623	1.2591
Nivolumab + Ipilimumab	0.7485	0.6033	0.9287

Table S2. Head-to-head indirect comparisons between ICI-based combination treatments. The abbreviations reported below are explained in Table 1.

DFS	HR	Lower.95	Upper.95	P
ARCC01 vs ARCC03	0.831	0.616	1.120	>0.05
ARCC01 vs TKI03	0.763	0.591	0.985	0.038
ARCC01 vs TKI05	0.823	0.615	1.102	>0.05
ARCC03 vs TKI03	0.918	0.715	1.179	>0.05
RCC03 vs TKI05	0.991	0.744	1.320	>0.05
TKI03 vs TKI05	1.079	0.848	1.374	>0.05

Table S3. Restricted mean survival time (RMST) of the five active treatments that showed a DFS benefit compared to placebo.

	RMST	Lower 95%CI	Upper 95%CI
CTRL	32.904	32.15	33.657
Pembrolizumab	36.089	34.697	37.482
Nivolumab + Ipilimumab	34.682	33.121	36.242
Sorafenib	34.334	33.215	35.454
Sunitinib	34.92	33.35	36.49

References (cited in the legends to Figures S1 and S2)

1. Gross-Goupil M, Kwon TG, Eto M, Ye D, Miyake H, Seo SI, Byun SS, Lee JL, Master V, Jin J, et al. Axitinib versus placebo as an adjuvant treatment of renal cell carcinoma: results from the phase III, randomized ATLAS trial. Ann Oncol. 2018;29(12):2371-2378. doi: 10.1093/annonc/mdy454.
2. Eisen T, Frangou E, Oza B, Ritchie AWS, Smith B, Kaplan R, Davis ID, Stockler MR, Albiges L, Escudier B, et al. Adjuvant Sorafenib for Renal Cell Carcinoma at Intermediate or High Risk of Relapse: Results From the SORCE Randomized Phase III Intergroup Trial. J Clin Oncol. 2020;38(34):4064-4075. doi: 10.1200/JCO.20.01800.
3. Haas NB, Manola J, Uzzo RG, Flaherty KT, Wood CG, Kane C, Jewett M, Dutcher JP, Atkins MB, Pins M, et al. Adjuvant sunitinib or sorafenib for high-risk, non-metastatic renal-cell carcinoma (ECOG-ACRIN E2805): a double-blind, placebo-controlled, randomised, phase 3 trial. Lancet. 2016;387(10032):2008-16. doi: 10.1016/S0140-6736(16)00559-6
4. Motzer RJ, Haas NB, Donskov F, Gross-Goupil M, Varlamov S, Kopyltsov E, Lee JL, Melichar B, Rini BI, Choueiri TK, et al. Randomized Phase III Trial of Adjuvant Pazopanib Versus Placebo After Nephrectomy in Patients With Localized or Locally Advanced Renal Cell Carcinoma. J Clin Oncol. 2017;35(35):3916-3923. doi: 10.1200/JCO.2017.73.5324.
5. Ravaud A, Motzer RJ, Pandha HS, George DJ, Pantuck AJ, Patel A, Chang YH, Escudier B, Donskov F, Magheli A, et al. Adjuvant Sunitinib in High-Risk Renal-Cell Carcinoma after Nephrectomy. N Engl J Med. 2016;375(23):2246-2254. doi: 10.1056/NEJMoa1611406
6. Ryan CW, Tangen CM, Heath EI, Stein MN, Meng MV, Alva AS, Pal SK, Puzanov I, Clark JI, Choueiri TK, et al. Adjuvant everolimus after surgery for renal cell carcinoma (EVEREST): a double-blind, placebo-controlled, randomised, phase 3 trial. Lancet. 2023;402(10407):1043-1051. doi: 10.1016/S0140-6736(23)00913-3.
7. Powles T, Tomczak P, Park SH, Venugopal B, Ferguson T, Symeonides SN, Hajek J, Gurney H, Chang YH, Lee JL, et al. Inves-tigators. Pembrolizumab versus placebo as post-nephrectomy adjuvant therapy for clear cell renal cell carcinoma (KEY-NOTE-564): 30-month follow-up analysis of a multicentre, randomised, double-blind, placebo-controlled, phase 3 trial. Lancet Oncol. 2022;23(9):1133-1144. doi: 10.1016/S1470-2045(22)00487-9.
8. Motzer RJ, Russo P, Grünwald V, Tomita Y, Zurawski B, Parikh O, Buti S, Barthélémy P, Goh JC, Ye D, et al. Adjuvant nivolumab plus ipilimumab versus placebo for localised renal cell carcinoma after nephrectomy (CheckMate 914): a double-blind, randomised, phase 3 trial. Lancet. 2023;401(10379):821-832. doi: 10.1016/S0140-6736(22)02574-0.
